# Nicotine withdrawal and agitation in ventilated critically ill patients

**DOI:** 10.1186/cc8954

**Published:** 2010-04-09

**Authors:** Olivier Lucidarme, Amélie Seguin, Cédric Daubin, Michel Ramakers, Nicolas Terzi, Patrice Beck, Pierre Charbonneau, Damien du Cheyron

**Affiliations:** 1 Service de Réanimation Polyvalente, CH mémorial France-Etats-Unis de Saint-Lô, 50000 Saint-Lô, France; 2 Service de Réanimation Médicale, CHU de Caen, 14033 Caen Cedex, France; 3 UPRES EA 2128, CHU de Caen, 14033 Caen Cedex, France

## Abstract

**Introduction:**

Smoking is highly addictive, and nicotine abstinence is associated with withdrawal syndrome in hospitalized patients. In this study, we aimed to evaluate the impact of sudden nicotine abstinence on the development of agitation and delirium, and on morbidities and outcomes in critically ill patients who required respiratory support, either noninvasive ventilation or intubation, and mechanical ventilation.

**Methods:**

We conducted a prospective, observational study in two intensive care units (ICUs). The 144 consecutive patients admitted to ICUs and requiring mechanical ventilation for >48 hours were included. Smoking status was assessed at ICU admission by using the Fagerström Test of Nicotine Dependence (FTND). Agitation, with the Sedation-Agitation Scale (SAS), and delirium, with the Intensive Care Delirium Screening Checklist (ICDSC), were tested twice daily during the ICU stay. Agitation and delirium were defined by SAS >4 and ICDSC >4, respectively. Nosocomial complications and outcomes were evaluated.

**Results:**

Smokers (*n* = 44) were younger and more frequently male and were more likely to have a history of alcoholism and to have septic shock as the reason for ICU admission than were nonsmokers. The incidence of agitation, but not delirium, increased significantly in the smoker group (64% versus 32%; *P *= 0.0005). Nicotine abstinence was associated with higher incidences of self-removal of tubes and catheters, and with more interventions, including the need for supplemental sedatives, analgesics, neuroleptics, and physical restraints. Sedation-free days, ventilator-free days, length of stay, and mortality in ICUs did not differ between groups. Multivariate analysis identified active smoking (OR, 3.13; 95% CI, 1.45-6.74; *P *= 0.003) as an independent risk factor for agitation. Based on a subgroup of 56 patients, analysis of 28 pairs of patients (smokers and nonsmokers in a 1:1 ratio) matched for age, gender, and alcoholism status found similar results regarding the role of nicotine withdrawal in increasing the risk of agitation during an ICU stay.

**Conclusions:**

Nicotine withdrawal was associated with agitation and higher morbidities in critically ill patients. These results suggest the need to look specifically at those patients with tobacco dependency by using the FTND in ICU settings. Identifying patients at risk of behavioral disorders may lead to earlier interventions in routine clinical practice.

## Introduction

Cigarette smoking is the main addiction in the world [1]. Tobacco use is associated with a high prevalence of alcohol and drug dependence, depression, and anxiety disorders [2,3]. Because the body develops a homeostatic response to nicotine, smokers have withdrawal symptoms on abstinence from the drug [1]. These symptoms peak during the first week of abstinence but sometimes are persistent for several weeks or months, and then gradually decrease to baseline levels [4,5]. In hospitalized patients, studies have reported several manifestations related to sudden nicotine abstinence, such as bradycardia, irritability, anxiety and agitation, confusion, or hallucinations, but intensive care unit (ICU) patients are usually excluded from published studies [6].

Behavioral disorders such as delirium and agitation in the critically ill occur with a high frequency, ranging from 15% to 80% of patients, and have been associated with increased morbidity and risk of mortality [7-13]. Many risk factors, such as history of hypertension and alcoholism, higher severity of acute disease, and clinical effects of sedative and analgesic agents, have been identified in the literature [12]. Few data exist in the literature regarding the behavioral impact of sudden nicotine abstinence in the ICU setting, except for one study, which identified smoking history as a risk factor for delirium in critically ill patients [8]. Moreover, nicotine-replacement therapy (NRT) remains a controversial topic in the ICU, and a retrospective study found an association between NRT and mortality [14].

Thus, we aimed to evaluate the nicotine-withdrawal syndrome in critically ill patients. We hypothesized that dependent smokers may have increased risk for agitation and delirium, and then increased morbidity, such as infections and accidental self-removal of tubes and catheters, related to these behavioral disorders.

## Materials and methods

### Patients

This prospective observational study was conducted over a period running from June 2007 to April 2008 in two adult ICUs (a 22-bed medical ICU in the University Hospital of Caen, Center 1, and an eight-bed medicosurgical ICU in the tertiary Memorial Hospital of Saint-Lô, Center 2, Normandy, France). All patients admitted to the ICUs and mechanically ventilated with either noninvasive ventilation or intubation for respiratory support for longer than 48 hours were considered eligible for the investigation. Patients were excluded if they were younger than 18 years or were determined to have a history of chronic dementia and psychosis, or acute neurologic diseases on admission, such as severe traumatic brain injury, ischemic stroke, or cerebral hemorrhage. NRT was forbidden during the study period. The study was approved by the local ethics committee. Patients were included after informed consent of the patient or next-of-kin was obtained.

### Data collection

The following demographic and clinical data were collected at ICU admission: age, gender, medical or surgical origin referring to the primary admission diagnosis, history of hypertension, chronic alcoholism and psychotropic therapy, smoking status, and primary diagnosis on admission to the ICUs. Alcohol consumption was considered chronic if it persisted for the whole year before admission, as defined by the National Institute on Alcohol Abuse and Alcoholism criteria for unhealthy alcohol use in the United States [15,16]. To assess the severity of the acute illness, the Simplified Acute Physiology Score II (SAPS II) [17] and the initial Sequential Organ Failure Assessment (SOFA) score [18] were determined within 24 hours after ICU admission. During the ICU stay, the duration of mechanical ventilation (either invasive or noninvasive ventilation), the cumulative dose and duration of drug exposure for sedation-analgesia, and the number of days per patient with heavy sedation, defined as a score ≥4 in the Ramsay sedation scale [19], were recorded. Finally, ICU length of stay and mortality were registered.

### Definitions

Tabagism was evaluated according to the tobacco load, which is quantified in pack-years, and the nicotine dependence, as assessed by the Fagerström Test of Nicotine dependence (FTND) [20] (Additional data file 1), obtained from patients or their closest relatives. Patient dependency was dichotomized in weak and strong by using a threshold value of 4 in this smoking scale. Patients were divided into two distinct groups: (1) smoker group, including patients with active smoking status; and (2) nonsmoker group, including patients with nonsmoking history or tobacco discontinuation for >6 months.

Agitation was assessed twice daily by nurses or physicians until ICU discharge, by using the modified Sedation-Agitation Scale (SAS) [21] (additional data file 2). SAS lists three levels of agitation. Patients were classified as "sedated" (SAS 1 to 3), "calm" (SAS 4), and "agitated" (SAS 5 to 7). Similarly, delirium was assessed for each patient twice daily by nurses or physicians until ICU discharge by using the Intensive Care Delirium Screening Checklist (ICDSC) score [22] (Additional data file 3). It includes eight items based on the *Diagnostic and Statistical Manual of Mental Disorders (DSM) IV *criteria [23] and features delirium, including inattention, disorientation, hallucination-delusion psychosis, psychomotor agitation or retardation, inappropriate speech or mood, sleep/wake-cycle disturbances, and symptom fluctuation. For each abnormal item, a score of 1 was given. Patients with an ICDSC score >4 were considered to be delirious. Sedated patients with altered level of consciousness of A or B on the ICDSC scale were not considered to have delirium. All degrees of agitation and delirium were then confirmed by an independent physician by using chart assessment.

Nosocomial infections were defined as follows: (1) ventilator-acquired pneumonia: clinical suspicion of pneumonia (that is, clinical and radiographic criteria), and at least one organism isolated by protective specimen brush at a concentration ≥10^3 ^colony-forming units (CFUs)/ml; (2) colonization of central venous catheters: at least one organism at a concentration ≥10^3 ^CFUs/ml identified by culture of the catheter tip with the Brun-Buisson technique [24]; (3) urinary catheter-related infection: the association of a leukocyturia at a concentration of ≥10^4^/ml with the presence of an organism at a concentration of 10^5 ^CFU/ml; (4) bacteremia: a positive hemoculture with the isolation of an organism or at least two positive hemocultures for a coagulase-negative *Staphylococcus*, according to the usual definitions [25].

### End points

Primary end points were either one or more agitation or delirium events during the ICU stay. Secondary end points were ventilator-free days (days alive and free from mechanical ventilation during the ICU stay); total dose of sedatives and analgesics administered during the ICU stay, including the extra doses needed to abort the episodes of agitation/delirium; and sedation-free days (days alive and free from sedatives and analgesics during the ICU stay); and complications related to agitation and delirium, such as requirement for supplemental sedation or physical restraints, use of additional antipsychotic medication; and self-extubation, self-removal of arterial, central venous and bladder catheters, nasogastric tubes, and nosocomial infections.

### Statistical analysis

With a 30% baseline incidence of smokers in the general population, we calculated that a sample size of at least 134 patients was necessary to achieve a 30% absolute difference in agitation/delirium incidence among smokers and nonsmokers at a beta error of 0.2 and an alpha error of 0.05. For univariate analysis, we used the χ^2 ^test for categoric variables with continuity correction when appropriate, the Fisher's Exact test for proportions, and the Mann-Whitney test for quantitative variables. To determine the set of independent predictors of agitation, a multivariate logistic regression analysis was performed by using backward stepwise selection. In the multivariate model, the outcome variable studied was agitation, defined as success if agitation occurred at least once. Variables with a *P *value < 0.1 in univariate analysis were included in the regression analysis, and then a *P *value of 0.2 was used to remove variables from the model. To avoid analyses that might have resulted in biased conclusions because of redundancy of the included variables, SAPS II and SOFA scores were not included in the same model. The performance of the final model on the test set was assessed by using the c-index and its 95% CI. To ensure that our findings were robust, we also performed a case-control approach nested in our study to assess the relative risk (RR) of agitation, according to the smoking status. We matched each smoker with each nonsmoker in a 1:1 ratio. Each pair had to fulfill three conditions: same age (± 5 years), same gender, and same status regarding alcohol consumption (defined as presence or absence of chronic alcoholism). We analyzed this subset of patients by using the Wilcoxon test, the MacNemar χ^2 ^test, and the pair-matched Mantel-Haenszel adjusted RR. Analysis was performed by using MedCalcSS version10.1 (MedCalc Software, Mariakerke, Belgium). The two-tailed significance level was set at *P *< 0.05.

## Results

### Patients

**Figure 1:**
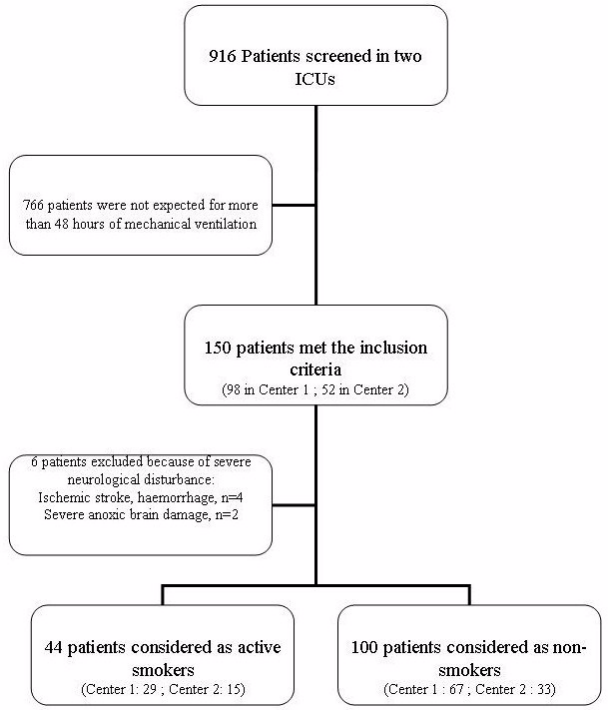
**Flow chart of the patients admitted to the intensive care units (ICUs) during the study period**. Center 1, Caen University Hospital; Center 2: Memorial St-Lô Hospital.

In total, 916 admissions were screened for enrollment. One hundred fifty consecutive patients met the inclusion criteria. Six patients were secondarily excluded, leaving 144 patients for analysis (Figure 1). Forty-four patients were classified as smokers, with a median FTND score of 5. Among them, 18 (41%) patients had a score ≤4 in the FTND scale and were considered to have weak dependency, whereas 26 (59%) patients had an FTND value >4 and were considered strongly dependent on nicotine. Each time it was possible, the Fagerström test was obtained from the patient at admission to the ICU or once recovery happened, if the test was initially obtained from next-of-kin. Overall, 38 patients were able to respond to the FTND, 26 at admission to ICU, and 12 after recovery. Both patient and next-of-kin responses were similar (median, 5.5 (4-7) versus 5 (4-7); *P *= 0.62, respectively), and in these cases, we have recorded the patient's test responses for analysis. Thus, only six (14%) FTND scores were obtained exclusively from next-of-kin, and were recorded as well.

### Mechanical ventilation

Regarding respiratory support, 45 (31%) patients in the overall cohort received noninvasive ventilation. In this subgroup of patients, the rate of intubation was 62% related to failure of noninvasive ventilation. Thus, only 17 patients (five smokers and 12 nonsmokers) were treated exclusively with noninvasive ventilation. Based on SAS and ICDSC scores, no statistical difference was recorded in the number of agitation events between both groups (data not shown).

### Smoking, agitation, and outcome

Table 1 presents the baseline characteristics of patients. Smokers were younger, and more of them were men, with more history of alcoholism and septic shock at ICU admission than had nonsmokers. As shown in Table 2, the frequency of agitation was higher in the smoker group (64% versus 32%; *P *= 0.0005), but delirium was not affected by smoking status. There was no central effect in the incidence of agitation. In a subgroup analysis comparing weak and strong tobacco dependencies according to FTND, no statistical difference was observed for the development of agitation (one or more episodes of agitation in nine patients among 18 with FTND ≤4, and in 19 patients among 26 with FTND >4, respectively; *P *= 0.2). Similarly, tobacco-load distribution did not differ between smokers with and without agitation during the ICU stay. In patients in whom agitation developed, the median number of days with agitation events was greater for smokers than for nonsmokers. The rates of accidental removal of tubes and catheters were higher for smokers, as well as the need for supplemental sedative and analgesic medications, physical restraints, and neuroleptics in comparison with nonsmokers (Table 2). The neuroleptic of choice was haloperidol, which was mainly instilled into the nasogastric tube. In five cases, patients received droperidol by infusion because of contraindications for nasogastric tubes. For supplemental sedation, propofol was the drug of choice, administered by conventional titration dosing and followed by infusion. For patients who were suspected to develop agitation/delirium during benzodiazepine or opiate withdrawal, bolus dosing and then higher doses of midazolam and morphine, respectively, were administered by infusion. Altogether, no differences among groups of smokers and nonsmokers were observed regarding type and dose of sedative medications in the treatment of the agitation and delirium events. Overall, the rate of nosocomial infections, the use and doses of sedatives and analgesics, sedation-free days and ventilator-free days, lengths of stay in ICU, and mortality rates did not differ among groups (Table 2).

**Table 1:** Baseline characteristics of patients

	Smokers (*n* = 44)	Nonsmokers (*n* = 100)	*P *value
Age, yr	54 (45.5-62.5)	69 (58-78.5)	<0.0001
Male gender, *n* (%)	40 (91)	70 (70)	0.006
Medical origin, *n* (%)	38 (86)	81 (81)	0.5
Source, admission from the ward, *n* (%)	9 (20)	29 (29)	0.3
Comorbidities, *n* (%)			
Hypertension	7 (16)	20 (20)	0.6
Alcoholism	30 (68)	20 (20)	<0.0001
Psychotropic drug use	15 (34)	25 (25)	0.3
Tobacco dependency			
Pack-years	28 (22-40)	NA	
Fagerström score	5 (3-6)	NA	NA
Score ≤4 (Weak dependence), *n* (%)	20 (45)		
Score >4 (Strong dependence), *n* (%)	24 (55)		
Primary diagnosis, *n* (%)			
Cardiac disease	1 (2)	16 (16)	
Respiratory disease	15 (34)	41 (41)	
Septic shock	14 (32)	18 (18)	0.007
Neurologic disease	6 (14)	7 (7)	
Surgery	2 (4)	14 (14)	
Other	6 (14)	8 (8)	
SAPS II	46.5 (39-60)	50 (40.5-63)	0.3
SOFA score	7.5 (5.5-10)	8 (6-10)	0.5
Vasopressor, *n* (%)	28 (64)	51 (51)	0.2
Body temperature >38.5°C, *n* (%)	12 (27)	17 (17)	0.2
PaO_2_/FiO_2_	222 (165-257)	186 (135-250)	0.1

Abbreviations: NA, not attributable; SAPS II, Simplified Acute Physiology Score II; SOFA, Sequential Organ Failure Assessment. Data are presented as number (percentage) or median (interquartile range).

**Table 2:** Morbidity and mortality according to the smoking status

	Smokers (*n* = 44)	Nonsmokers (*n* = 100)	*P *value
Evaluation of agitation (SAS ≥5)			
Patients who develop at least one event, *n* (%)	28 (64)	32 (32)	0.0005
Days with agitation/patient	1.5 (0-4)	0 (0-1)	0.0006
Number of episodes of agitation/day/patient with agitation	2 (1-3.5)	2 (1-4)	0.9
Evaluation of delirium (ICDSC ≥4)			
Patients who develop at least one event, *n* (%)	16 (37)	24 (24)	0.2
Rates of nosocomial infections^$^			
Ventilator-associated pneumonia	16.7	15.9	0.5
Urinary tract infection	3.9	2.9	0.4
Catheter colonization	29.0	26.4	0.1
Bacteremia	4.1	3.9	0.6
Rates of accidental removal of tubes and catheters*			
Endotracheal tube (self-extubation)	7.5	3.7	<0.0001
Arterial, venous, or bladder catheter	20.1	11.9	<0.0001
Nasogastric tube	33.5	22.4	0.0003
Sedation/analgesia			
Number of days/patient with Ramsay score ≥4	3 (2-5)	4 (2-5.5)	0.4
Sedation-free days, days	1.5 (0-4)	2 (0-4.5)	0.6
Total dose of midazolam (mg/kg)^§^	3 (0-14)	3 (0-10)	0.6
Total dose of propofol (mg/kg)^§^	42 (9-122)	18 (0-79)	0.1
Total dose of sufentanyl (μg/kg)^§^	12 (2-25)	9 (4-22)	0.9
Sedation/physical restraints related to agitation/delirium, *n* (%)			
Supplemental sedatives and analgesics	23 (52)	15 (15)	<0.0001
Physical restraints	21 (48)	13 (13)	<0.0001
Neuroleptics	11 (25)	7 (7)	0.005
Mechanical ventilation (*n* = 100%)			
Noninvasive ventilation (NIV), *n* (%)	14 (32)	31 (31)	0.9
Failure of NIV requiring intubation, *n* (% of NIV)	9 (64)	12 (39)	0.2
Length of NIV, days	3 (2-7)	2 (1-3.5)	0.07
Length of mechanical ventilation, days	10 (4.5-18.5)	10 (5.5-19.5)	0.6
Mechanical ventilation-free days, days	1.5 (0.5-4)	2 (0-4)	0.7
ICU LOS, days	15 (6-22.5)	14 (8-27.5)	0.8
ICU mortality, *n* (%)	7 (16)	28 (28)	0.1

Abbreviations: SAS = Sedation-Agitation Scale; ICDSC = Intensive Care Delirium Screening Checklist; NIV = noninvasive ventilation; ICU = Intensive Care Unit; LOS = length of stay. Data are presented as number (percentage) or median (interquartile range) when appropriate. ^$ ^Rates of nosocomial infections according to the time at risk for infection (number of ventilator-associated pneumonias, catheter colonizations, and urinary tract infections per 1,000 ventilation-days, 1,000 catheter-days, and 1,000 bladder catheter-days, respectively). Number of events of bacteremia for 1,000 days if ICU stay. *Rates of accidental removal of endotracheal tubes (self-extubation), venous, arterial, and bladder catheters, nasogastric tubes, according to the time at risk for removal (number of tubes or catheters removed per 1,000 days in place). ^§^Total dose of sedatives and analgesics administered during ICU stay, taking into account the extra doses needed to abort the episodes of agitation/delirium.

### Risk factors for agitation

By univariate analysis, the risk factors associated with agitation were age, male gender, SOFA score, and active smoking (Table 3). After adjustment for age, male gender, chronic alcoholism, SAPS II, and PaO_2_/FiO_2 _ratio, multivariate analysis identified active smoking as an independent predictor of agitation (OR, 3.13; 95% CI, 1.45 to 6.74) (Table 3). The discriminatory performance of the composite logistic model demonstrated a good c-index of 0.701 (95% CI, 0.624 to 0.779) for agitation. Of note, of the 44 smokers, 30 had a history of chronic alcoholism (*P *< 0.0001, χ^2 ^test; Table 1). Chronic alcoholism, however, reached a level of borderline significance for agitation only with univariate analysis (OR, 1.91; 95% CI, 0.95 to 3.83; *P *= 0.07), and then was not identified as independent predictor for agitation in the multivariate analysis.

**Table 3:** Univariate and multivariate analysis of factors associated with agitation in the ICU

	Univariate analysis		Multivariate analysis	
	**Odd ratio (95%CI)**	***P *value**	**Odd ratio (95%CI)**	***P *value**

Age*	0.97 (0.95-0.99)	0.02	-	-
Male gender	2.91 (1.21-6.99)	0.02	2.27 (0.90-5.68)	0.08
Hypertension	1.25 (0.88-1.60)	0.3		
Chronic alcoholism*	1.91 (0.95-3.83)	0.07	-	-
Chronic use of psychotropics	1.39 (0.67-2.90)	0.4		
Septic shock	0.96 (0.64-1.45)	0.9		
SAPS II	0.98 (0.96-1.01)	0.07	0.98 (0.96-1.01)	0.1
SOFA^§^	0.88 (0.79-0.98)	0.02		
Body temperature >38.5°C	1.22 (0.82-1.57)	0.2		
PaO_2_/FiO_2_	0.81 (0.56-1.04)	0.09	-	-
Active smoking	3.72 (1.77-7.83)	0.0005	3.13 (1.45-6.74)	0.003

Abbreviations: 95%CI = 95% confidence interval; SAPS II = Simplified Acute Physiology Score II; SOFA = Sequential Organ Failure Assessment. *Age and Chronic alcoholism were entered in the model and then removed from the model by the backward stepwise selection. ^§^Despite statistical significance in univariate analysis, SOFA was not included in the multivariate model to avoid biased conclusions due to redundancy of the variables that are part of both SAPS II and SOFA.

Matching was possible in 62 patients representing 43% of the patients considered in the main analysis (Table 4). By matching analysis, the rate of agitation increased from 42% in matched controls to 80% among cases. A total of 10 and three pairs of patients yielded the presence and absence of agitation, respectively. Fifteen and three pairs of patients were associated with the presence of agitation in smokers and nonsmokers, respectively. The number of conflicting pairs was statistically significant (χ^2 ^= 8.2; *P *= 0.004), and tobacco dependency was associated with an increased risk for agitation compared with a no-smoking history (RR, 1.9; 95% CI, 1.3 to 3.0; *P *= 0.004).

**Table 4:** Characteristics of the 56 patients included in the matched case-control analysis

	Smokers (*n* = 31)	Nonsmokers (*n* = 31)	*P *Value
Age,^$ ^years	56 (51-64)	58 (55-66)	NA
Male gender,^$ ^*n* (%)	30 (97)	30 (97)	NA
Medical origin, *n* (%)	26 (84)	23 (74)	0.5
Source, admission from the ward, *n* (%)	6 (19)	8 (26)	0.8
Comorbidities, *n* (%)			
Hypertension	5 (16)	8 (26)	0.5
Alcoholism	20 (65)	20 (65)	NA
Psychotropic drug use	10 (32)	7 (23)	0.6
Tobacco dependency			
Pack-years	31 (22-39)	NA	
Fagerström score	6 (3.5-6.5)	NA	
Score ≤4 (weak dependence), *n* (%)	13 (42)		
Score >4 (strong dependence), *n* (%)	18 (64)		
Primary diagnosis,^$ ^*n* (%)			
Cardiac disease	1 (4)	4 (13)	
Respiratory disease	11 (35)	13 (42)	0.2
Septic shock	10 (32)	7 (23)	
Neurologic disease	2 (6)	2 (6)	
Surgery	1 (4)	4 (13)	
Other	6 (19)	1 (4)	
SAPS II	48 (38-59)	51 (40-61)	0.8
SOFA	8 (5-10)	8 (6-11)	0.4
Vasopressor, *n* (%)	23 (74)	20 (65)	0.6
Body temperature >38.5°C, *n* (%)	13 (42)	16 (52)	0.6
NIV, *n* (%)	10 (32)	9 (29)	0.9
Failure of NIV, *n* (% of NIV)	7 (70)	7 (78)	0.9
PaO_2_/FiO_2 _ratio	205 (165-260)	189 (146-255)	0.6
Sedation-analgesia			
Number of days/patient with Ramsay score ≥4	3 (2-5)	3 (2-6)	0.5
Sedation-free days, days	1 (0-4)	3 (0-6)	0.2
Total dose of midazolam (mg/kg)^§^	2 (0-11)	2 (0-8)	0.8
Total dose of propofol (mg/kg)^§^	48 (11-130)	7 (0-89)	0.3
Total dose of sufentanyl (μg/kg)^§^	13 (2-24)	10 (2-45)	0.7
At least one event of agitation,* *n* (%)	25 (80)	13 (42)	0.004
At least one event of delirium, *n* (%)	11 (35)	7 (23)	0.4
ICU LOS, days	13 (6-23)	15 (7-30)	0.9
ICU mortality, *n* (%)	5 (16)	8 (26)	0.5

Results are expressed as number (%) or median (interquartile range) when appropriate. ^$^Matched variables. ^§^Total dose of sedatives and analgesics administered during ICU stay, taking into account the extra doses needed to abort the episodes of agitation/delirium. Abbreviations: NA = not attributable; SAPS II = Simplified Acute Physiology Score II; SOFA = Sequential Organ Failure Assessment; NIV = noninvasive ventilation; ICU = intensive care unit; LOS = length of stay.

## Discussion

This prospective observational study is one of the first to focus specifically on the impact of sudden nicotine abstinence in the ICU setting. Nicotine withdrawal was associated with agitation, but not with delirium, and smokers had a greater frequency of adverse events, such as accidental self-removal of tubes and catheters, and new interventions including supplemental sedation and physical restraint related to agitation events.

### Incidence

As to external validity, the 31% of smokers we observed in our cohort study, among them a majority of male gender, reflects the proportion of smokers that are usually reported in the general population [1]. Moreover, the 42% incidence of agitation in this study is in agreement with those previously reported in critically ill patients [13,26-28]. Among these publications that focused on agitation in the ICU setting, only one included smoking status as a variable of interest [13], which was, however, not identified as a risk factor for agitation. This discrepancy may reflect differences in study design, case-mix population (for example, medicosurgical patients versus medical patients), screening instrument (SAS versus Motor Activity Assessment Scale [29]), and local sedation protocols.

### Agitation

The presence of agitation in an acutely ill patient requiring mechanical ventilation can be a potentially life-threatening problem. Agitation has been associated with physiologic changes producing interference with mechanical ventilation, increased oxygen consumption, and failure to cooperate with treatment [21]. In our study, smoking abstinence, even in patients with weak tobacco dependency, was associated with agitation and then self-removal of catheters and tubes, as already reported [13,26,28]. Nicotine withdrawal as risk factor for agitation was also suggested by the results that emerged from analyses nested in our trial. The risk of agitation increased after adjustment for potential confounders. The consistency of the results between the two statistical methods used for adjustment indicates their robustness to assumptions and thus produces more confidence in their validity.

The adequate strategy for dealing with agitation is not clearly established in ICU. Patients with agitation were, however, more likely to be given supplemental sedative and analgesic agents or physical restraints to prevent self-inflicted injuries and thus treatment interference [13,27,28]. Physical restraints could promote, however, the occurrence of agitation, as described in psychiatric emergency departments [30]. The difficulty of supporting the use of this therapeutic is also related to legal and ethical points of view. Restraint is a complex topic and should be discontinued as soon as the patient has no further indication for its use [31]. This may explain the increased prescription of antipsychotics we observed in our smoker group, although no formal protocol governed their use in both ICUs. In a retrospective cohort study focusing on mechanically ventilated patients [32], haloperidol use was associated with lower hospital mortality, at least in part because of a reduced use of sedatives and analgesics. Haloperidol has, however, a number of side effects, the most problematic of which is prolongation of the corrected QT interval [33]. A large randomized trial testing the best strategy to prevent and treat agitation is warranted, and either nicotine-replacement therapy [34,35] or drugs such as clonidine [36] and dexmedetomidine [37] may be promising treatments in this situation.

### Delirium

The 28% incidence of delirium we recorded at the low level of the wide range cited in the literature (13% to 70%) [8,11,38-40]. Heterogeneity between studies may also reflect differences in patient selection, delirium scales, and practices, including how sedation is administered, among institutions. Nicotine withdrawal has been suggested as an underrecognized cause of delirium in patients with acute brain injury [41]. In contrast to the finding of Dubois and colleagues [8], we found no association between nicotine withdrawal and delirium. This discrepancy may, however, be explained partly by the small size of our cohort, the exclusion of acute neurologic diseases in our study, the absence of a standardized protocol for sedation and antipsychotic medications, and the difficulties of standardizing the recording of the ICDSC items by both nurses and physicians. As reported by others in trauma patients [39] and mechanically ventilated medical patients [40], using another tool (for example, CAM-ICU [9]) and categorizing as either hypoactive or hyperactive delirium might have elicited the delirium incidence and then found a possible association between smoking and hyperactive delirium.

### Limits of the study

Some study limitations must be addressed. First, objective measurement of tobacco addiction, like urinary cotinine, was not performed, and conclusions drawn from this observational study in a small number of patients cannot be generalized. The lack of any association between the level of addiction and either agitation rate or most important outcomes (length of stay, organ failure, and mortality) may also be the result of the small sample size. Second, tobacco-smoking status and end points were not collected in a blinded manner by nurses and staff, and this point may be considered a potential bias. Third, the use of sedative and analgesic drugs, as well as discontinuation of physical restraints, were left to the attending physician's judgment, and thus, were likely to be somewhat random. As a consequence, "organ failure" of the brain (delirium and agitation) may have been over- or underestimated in some cases. And fourth, adjusted analyses can potentially yield distorted associations by selection or omission of variables that influence outcome. Regarding age, gender, alcoholism, and septic shock, a large imbalance between smokers and nonsmokers was observed in our cohort study and may be considered as potential bias. These confounding factors are, however, taken into account in the matched analysis that confirms our finding.

## Conclusions

In mechanically ventilated patients, sudden nicotine abstinence was associated with severe agitation and its consequences, such as self-removal of tubes and catheters. These results suggest the need to be aware of nicotine-withdrawal syndrome in critically ill patients, and then support the necessity to improve strategies to prevent and treat agitation earlier. Based on these findings, the use of nicotine-replacement therapy should be tested by a well-designed, randomized controlled clinical trial in the ICU setting.

## Key messages

• Agitation is a common event in critically ill patients.

• Nicotine withdrawal was identified as risk factor of agitation, but not delirium, in multivariate and matched case-control analyses adjusted for confounding factors.

• Agitation was associated with a higher adverse-event rate, such as accidental self-removal of tubes and catheters, and new interventions including supplemental sedation and physical restraint.

• These results suggest the need to be aware of nicotine-withdrawal syndrome in critically ill patients, and then to support the necessity to improve strategies to prevent and treat agitation earlier.

## Competing interests

The authors declare that they have no competing interests.

## Authors' contributions

OL and DdC initiated the study and the design. DdC was involved in the interpretation of the results and wrote the manuscript. OL helped to draft the manuscript. AS, CD, MR, NT, PB and PC contributed to the conception of the study. All authors read and approved the final manuscript.

## Authors' information

This work was presented in part at the 37th Congress of the Société de Réanimation de Langue Française (SRLF) held in January 2009 in Paris, France.

## Supplementary Material

Additional file 1 Fagerström Test for Nicotine Dependence (FTND). Click here for file

Additional file 2 Riker Sedation-Agitation Scale (SAS). Click here for file

Additional file 3 Intensive Care Delirium Screening Checklist (ICDSC). Click here for file
