# Supplementary material for: Nicotine withdrawal and agitation in ventilated critically ill patients
Source: Crit Care. 2010 Apr 9;14(2):R58. doi: 10.1186/cc8954 (PMC2887179; doi:10.1186/cc8954)
Supplement: Additional file 1 — Fagerström Test for Nicotine Dependence (FTND). [file cc8954-S1.DOC]

**Additional file 1: Fagerström Test for Nicotine Dependence (FTND)**

From [20].
