# Supplementary material for: Nicotine withdrawal and agitation in ventilated critically ill patients
Source: Crit Care. 2010 Apr 9;14(2):R58. doi: 10.1186/cc8954 (PMC2887179; doi:10.1186/cc8954)
Supplement: Additional file 2 — Riker Sedation-Agitation Scale (SAS). [file cc8954-S2.DOC]

**Additional file 2: Riker Sedation-Agitation Scale (SAS)**

From [21]
