# Supplementary material for: Nicotine withdrawal and agitation in ventilated critically ill patients
Source: Crit Care. 2010 Apr 9;14(2):R58. doi: 10.1186/cc8954 (PMC2887179; doi:10.1186/cc8954)
Supplement: Additional file 3 — Intensive Care Delirium Screening Checklist (ICDSC). [file cc8954-S3.DOC]

**Additional file 3: Intensive Care Delirium Screening Checklist (ICDSC)**

^a^ if A or B, then no other items are assessed that day.

From [22]
